# Supplementary material for: Distinct Prostate Cancer Survival Outcomes in Firefighters: A Population-Based Study
Source: Cancers (Basel). 2024 Mar 27;16(7):1305. doi: 10.3390/cancers16071305 (PMC11010940; doi:10.3390/cancers16071305)
Supplement: Supplementary file 1 [file cancers-16-01305-s001.zip › cancers-2868358-supplementary.pdf]

Supplementary tables

<table>
<tr><td colspan="7">Supplementary Table S1.
Baseline characteristics of prostate cancer in localized stage (all ages) and of regional/distant stages (<65 and ≥ 65 years of age) and by firefighters, Florida 2004-2014.</td></tr>
<tr><th rowspan="2">Localized Stage</th><th colspan="6">All Patients</th></tr>
<tr><th colspan="2">Total</th><th colspan="2">Firefighters</th><th colspan="2">Non-Firefighters</th></tr>
<tr><td>N</td><td colspan="2">123,043</td><td colspan="2">896</td><td colspan="2">122,147</td></tr>
<tr><td>Median Age (years)</td><td colspan="2">67</td><td colspan="2">63</td><td colspan="2">67</td></tr>
<tr><td>Mean PSA (ng/mL)</td><td colspan="2">9.6</td><td colspan="2">8.2</td><td colspan="2">9.6</td></tr>
<tr><td>Gleason score distribution (%)</td><td colspan="6"></td></tr>
<tr><td>≤6</td><td colspan="2">47.7</td><td colspan="2">52.2</td><td colspan="2">47.7</td></tr>
<tr><td>7</td><td colspan="2">36.5</td><td colspan="2">34.6</td><td colspan="2">36.5</td></tr>
<tr><td>≥8</td><td colspan="2">5.8</td><td colspan="2">7.0</td><td colspan="2">5.7</td></tr>
<tr><td>Unknown</td><td colspan="2">10.1</td><td colspan="2">6.1</td><td colspan="2">10.1</td></tr>
<tr><td>Receipt of Surgery (%)</td><td colspan="2">36.6</td><td colspan="2">44.4</td><td colspan="2">36.5</td></tr>
<tr><td>Receipt of Radiotherapy (%)</td><td colspan="2">49.2</td><td colspan="2">45.2</td><td colspan="2">49.2</td></tr>
<tr><th rowspan="2">Regional/Distant Stage</th><th colspan="3">Younger than 65 years old</th><th colspan="3">65 years old and older</th></tr>
<tr><th>Total</th><th>Firefighters</th><th>Non-Firefighters</th><th>Total</th><th>Firefighters</th><th>Non-Firefighters</th></tr>
<tr><td>Regional (n)</td><td>6,165</td><td>69</td><td>6,096</td><td>5,760</td><td>22</td><td>5,738</td></tr>
<tr><td>Distant (n)</td><td>1,936</td><td><10*</td><td>1,929</td><td>4,457</td><td>17</td><td>4,440</td></tr>
<tr><td>Median Age (years)</td><td>59</td><td>57</td><td>59</td><td>71</td><td>73</td><td>71</td></tr>
<tr><td>Mean PSA (ng/mL)</td><td>24.8</td><td>12.6</td><td>24.9</td><td>34.1</td><td>31.2</td><td>34.1</td></tr>
<tr><td>Gleason score distribution (%)</td><td></td><td></td><td></td><td></td><td></td><td></td></tr>
<tr><td>≤6</td><td>19.5</td><td>26.3</td><td>19.4</td><td>11.7</td><td>15..4</td><td>11.7</td></tr>
<tr><td>7</td><td>49.9</td><td>48.7</td><td>49.9</td><td>40.2</td><td>30.8</td><td>40.2</td></tr>
<tr><td>≥8</td><td>16.1</td><td>19.7</td><td>16.1</td><td>20.3</td><td>28.2</td><td>20.2</td></tr>
<tr><td>Unknown</td><td>14.5</td><td>5.3</td><td>14.6</td><td>27.9</td><td>25.6</td><td>27.9</td></tr>
<tr><td>Receipt of Surgery (%)</td><td>70.6</td><td>92.1</td><td>70.4</td><td>46.5</td><td>51.3</td><td>46.5</td></tr>
<tr><td>Receipt of Radiotherapy (%)</td><td>21.0</td><td>17.1</td><td>21.0</td><td>24.4</td><td>23.1</td><td>24.4</td></tr>
<tr><td colspan="7">*: Proportions not shown as N is less than 10; PSA: prostatic-specific antigen.</td></tr>
</table>

Supplementary Table S2.
Multivariable Cox regression: determinants of survival for Localized stage for firefighters and non-firefighters, Florida 2004-2014, excluding treatment variables.

Variable	Category	Multivariable Model for Localized Stage	
		aHR (95%CI)	P-value
**Occupation**	Non-Firefighters	Ref	
	Firefighters	0.52 (0.33, 0.80)	0.003
**Year of diagnosis**	2004-2009	Ref	<.001
	2010-2014	0.78 (0.73, 0.84)	
**Age at diagnosis (years)**	18-54	Ref	<.001
	55-64	1.44 (1.25, 1.67)	<.001
	65 - 74	2.13 (1.85, 2.45)	<.001
	75 - 84	3.68 (3.20, 4.23)	
**Race**	White	Ref	0.201
	Black	1.05 (0.97, 1.13)	0.003
	Other/Unknown	0.75 (0.62, 0.91)	
**Ethnicity**	Non-Hispanic	Ref	
	Hispanic	1.02 (0.93, 1.11)	0.650
	Unknown	0.70 (0.50, 1.00)	0.049
**Insurance**	Uninsured/Unknown	Ref	
	Insured	0.88 (0.79, 0.99)	0.032
**SES**	20% - 100% poverty	Ref	
	10% - <20% poverty	0.91 (0.84, 0.98)	0.019
	5% - <10% poverty	0.84 (0.77, 0.91)	<.001
	0% - <5% poverty	0.79 (0.72, 0.87)	<.001
	Unknown	0.66 (0.60, 0.74)	<.001
**Cigarette use**	Never	Ref	
	History	1.60 (1.47, 1.75)	<.001
	Current	1.13 (1.06, 1.21)	<.001
	Unknown	1.20 (1.13, 1.29)	<.001
**Grade**	Well-differentiate	Ref	
	Moderately differentiated	0.94 (0.78, 1.15)	0.565
	Poorly differentiated	1.29 (1.05, 1.58)	0.017
	Undifferentiated	2.12 (1.56, 2.88)	<.001
	Unknown/not stated	1.11 (0.88, 1.39)	0.38
**PSA**	<10ng/mL	Ref	
	10-20ng/mL	1.60 (1.48, 1.72)	<.001
	>20ng/mL	2.36 (2.19, 2.55)	<.001
	Unknown	1.51 (1.41, 1.62)	<.001
**Gleason score**	≤6	Ref	
	7	1.77 (1.61, 1.96)	<.001
	≥8	4.22 (3.70, 4.81)	<.001
	Unknown	2.26 (2.01, 2.53)	<.001

Ref: Reference; SES: Socio-Economic Status; SES: Socioeconomic Status reported as the percent poverty level of the patients' neighborhood at the time of cancer diagnosis. PSA: Prostatic-Specific Antigen; SD: Standard Deviation; Min: Minimum; Max: Maximum; HR: Hazard Ratio; aHR: Adjusted Hazard Ratio; 95%CI: 95% confidence interval.
